# Human herpesviruses-encoded dUTPases: a family of proteins that modulate dendritic cell function and innate immunity

**DOI:** 10.3389/fmicb.2014.00504

**Published:** 2014-09-26

**Authors:** Maria Eugenia Ariza, Ronald Glaser, Marshall V. Williams

**Affiliations:** ^1^Department of Molecular Virology, Immunology, and Medical Genetics, The Ohio State University College of MedicineColumbus, OH, USA; ^2^Institute for Behavioral Medicine Research, The Ohio State University College of MedicineColumbus, OH, USA

**Keywords:** dUTPase, dendritic cells, TLR2, human herpesviruses, cytokines

## Abstract

We have previously shown that Epstein-Barr virus (EBV)-encoded dUTPase can modulate innate immune responses through the activation of TLR2 and NF-κB signaling. However, whether this novel immune function of the dUTPase is specific for EBV or a common property of the *Herpesviridae* family is not known. In this study, we demonstrate that the purified viral dUTPases encoded by herpes simplex virus type 2 (HSV-2), human herpesvirus-6A (HHV-6A), human herpesvirus-8 (HHV-8) and varicella-zoster virus (VZV) differentially activate NF-κB through ligation of TLR2/TLR1 heterodimers. Furthermore, activation of NF-κB by the viral dUTPases was inhibited by anti-TLR2 blocking antibodies (Abs) and the over-expression of dominant-negative constructs of TLR2, lacking the TIR domain, and MyD88 in human embryonic kidney 293 cells expressing TLR2/TLR1. In addition, treatment of human dendritic cells and PBMCs with the herpesviruses-encoded dUTPases from HSV-2, HHV-6A, HHV-8, and VZV resulted in the secretion of the inflammatory cytokines IL-1β, IL-6, IL-8, IL-12, TNF-α, IL-10, and IFN-γ. Interestingly, blocking experiments revealed that the anti-TLR2 Ab significantly reduced the secretion of cytokines by the various herpesviruses-encoded dUTPases (*p* < 0.05). To our knowledge, this is the first report demonstrating that a non-structural protein encoded by herpesviruses HHV-6A, HHV-8, VZV and to a lesser extent HSV-2 is a pathogen-associated molecular pattern. Our results reveal a novel function of the virus-encoded dUTPases, which may be important to the pathophysiology of diseases caused by these viruses. More importantly, this study demonstrates that the immunomodulatory functions of dUTPases are a common property of the *Herpesviridae* family and thus, the dUTPase could be a potential target for the development of novel therapeutic agents against infections caused by these herpesviruses.

## Introduction

The innate immune response is an early line of defense that is essential for the detection of viruses. Cells contain a variety of sensors referred to as pattern recognition receptors (PRRs) that detect pathogen associated molecular patterns (PAMPs). The primary PRRs recognizing virus PAMPs include the retinoic acid-inducible gene 1 (RIG-I)-like receptors (RLR), the nucleotide oligomerization domain (NOD)-like receptors (NLRs) and the Toll-like receptors (TLRs) (Kawai and Akira, 2006; Gilliet et al., 2008). Monocytes/macrophages and dendritic cells (DCs) are professional antigen presenting cells (APCs) and play an important role in regulating the balance between tolerance and immune responses that initiate innate and adaptive immunity. There is accumulating evidence demonstrating that human herpesviruses infect monocytes, macrophages and DCs resulting in non-productive, productive or latent infections (Kondo et al., 1991; Blasig et al., 1997; Hahn et al., 1998; Savard et al., 2000; Abendroth et al., 2001; Mikloska et al., 2001; Zhang et al., 2001; Kakimoto et al., 2002; Li et al., 2002; Morrow et al., 2003; Senechal et al., 2004; Smith et al., 2005; Rappocciolo et al., 2006; Walling et al., 2007; Goldwich et al., 2011; Wang et al., 2012), and alter their functions (Kruse et al., 2000; Niiya et al., 2004; West et al., 2011; Gregory et al., 2012; Gustafsson et al., 2013; Stefanidou et al., 2013). However, the mechanism(s) by which human herpesviruses alter macrophage/DC function is unclear. Likewise, studies to determine the mechanism(s) by which the innate immune system is activated by herpesviruses have focused primarily on the intracellular recognition pathway for the CG rich DNA of these viruses or on the extracellular recognition of a viral-encoded structural protein(s) by TLR2 (Compton et al., 2003; Kurt-Jones et al., 2004; Aravalli et al., 2005; Wang et al., 2005; Boehme et al., 2006; Sato et al., 2006; Gaudreault et al., 2007; Paludan et al., 2011; Cal et al., 2012; Leoni et al., 2012). However, to the best of our knowledge there have not been any studies to determine whether non-structural proteins encoded by the human herpesviruses act as PAMPs and alter DC function.

The *Herpesviridae* family, which contains several members that are pathogenic for humans, are divided into three subfamilies (α, β, and γ) based upon their cellular tropism and genomic structure. Viruses from the three subfamilies contain a subset of genes that by criteria of genomic position and similarities in encoded amino acids sequences are common to all members. One such gene encodes for a deoxyuridine triphosphate nucleotidohydrolase (dUTPase). dUTPases represent a family of metalloenzymes that catalyze the hydrolysis of dUTP to dUMP and pyrophosphate (Nyman, 2001). dUTPases are divided into three subgroups based upon their structure and specificity for dUTP. The monomeric dUTPases, which are thought to have arisen from the trimeric dUTPases by gene duplication (Baldo and McClure, 1999), are found exclusively in herpesviruses (McGeehan et al., 2001). We have recently shown that the Epstein-Barr virus (EBV)-encoded dUTPase, which is an early protein expressed during lytic/abortive-lytic replication of the virus, possesses novel functions in innate immunity due in part to the activation of toll-like receptor TLR2 and subsequent modulation of downstream genes involved in type I interferon (IFNα/β) and cytokine/chemokine receptor signaling pathways (Glaser et al., 2006; Waldman et al., 2008; Ariza et al., 2009, 2013). While the members of the human herpesvirus family have considerable diversity with respect to cellular tropism and pathogenesis, they all encode for putative dUTPases, suggesting that these proteins may be critical to the biology of these viruses. Furthermore, it is important to understand what virus-encoded macromolecules have the potential to modulate the innate and adaptive immune systems. In this report, we provide compelling evidence demonstrating that the immune modulatory properties of the monomeric dUTPases are not restricted to EBV, but rather it is a property of several members of the human herpesviruses.

## Materials and methods

### Reagents

The NF-κB luciferase promoter construct pNF-κB-Luc and the transfection control reporter vector pRL-TK, were purchased from Clontech Laboratories, Inc., (Mountain View, CA), and Promega (Madison, WI), respectively. Wild-type pCMV-TLR1 vector was a gift from Dr Koichi Kuwano (Kurume University School of Medicine, Kurume, Japan). pCMV-MyD88DN expression construct was a gift from Dr. Jason A. Boch (Department of Medicine, Harvard Medical School, Boston, MA). Dominant-negative pZero-hTLR2 expression plasmid, puromycin, blasticidin, zymosan, FSL-1, and Pam3Csk4 were purchased from Invivogen (San Diego, California). IgG2a Isotype control monoclonal antibody was purchased from eBioscience (San Diego, California) and anti-TLR2 (clone TL2.1) monoclonal antibody was purchased from Imgenex (San Diego, California).

### Cloning of the herpesviruses dUTPases

Subcloning of the dUTPase genes from human herpesvirus-6A (HHV-6A, U45; kindly provided by Dr. Dharam Ablashi, HHV-6 Foundation), human herpesvirus-8 (HHV-8, ORF54; clone L54, which was obtained through the NIH AIDS Reagent Program, Division of AIDS, NIAID, NIH from Drs. Patrick Moore and Yan Chang), herpes simplex virus 2 (HSV-2, UL50; pGEM-HSV2dUT; kindly provided by Dr. Salvatore Caradonna, Rowan University), varicella-zoster virus (VZV, ORF8) (Ross et al., 1997), as well as subcloning of the human dUTPase (kindly provided by Dr. Evan McIntosh) (McIntosh et al., 1992) was conducted by PCR amplification using the forward and reverse primer sets (125 pmol of each) specific for each gene, DNA (140 ng), high fidelity PCR supermix (Invitrogen) and the following PCR conditions: Denaturation at 94°C for 3 min (') (1 cycle) followed by 35 cycles of 94°C for 30 s (sec), 50°C for 30 s, 72°C for 1′ and one cycle at 72°C for 20′. The specific PCR primer sequences used were HHV6AdUT: Forward (F): 5′-GGATCCATGTACAGCGCAATTTCAG-3′, Reverse (R): 5′-AAGCTTTTAAGCGTTATTGGAGGC-3′; HHV8dUT: F: 5′-CTTGGATCCATGAACAACCGCCGAGGC-3′, R: 5′-CCGGTTGAATTCTTAC TAAAACCCAGA-3′; HSV2dUT: F: 5′-CTCGAGATGAGTCAGTGGGGGCCCAGGGC-3′, R: 5′-GAATTCCTAGATGCCAGTGGAGCCAAACC-3′; VZVdUT: F: 5′-CTCGAGATTGATCCCATCTT GGAAACGGC-3′, R: 5′-GAATTCTTAATGTTTTAGTAGAAAATCGAC-3′; and HudUT: F: 5′-AAGCTTGGATCCATGCAGCTCCGCTTTGC-3′, R: 5′-TCCACTGGAAAGAATTAAGAATTCGT CGAC-3′. The PCR product was purified using the QIAquick gel extraction kit (QIAGEN) and cloned into the protein expression vector pTrcHis Topo (Invitrogen). Twenty individual clones for each dUTPase construct type were isolated following transformation of *E. coli* Top 10 competent cells, DNA was then purified using the QIAPrep Spin Miniprep kit (QIAGEN), screened by PCR for the presence of specific dUTPase genes and the sequence verified by DNA sequencing analysis. The pTrcHisdUT constructs, containing the specific dUTPase gene of interest in the correct orientation and in frame, were used to transform *E. coli* BL21(DE3)*plyS* competent cells for purification of recombinant proteins as described below.

### Purification of recombinant herpesviruses' dUTPase proteins

The recombinant herpesviruses' dUTPase proteins as well as the human dUTPase protein were purified using HisPur™ Spin columns (3 ml resin bed) as described by the manufacturer (Pierce, Rockford, IL). Briefly, BL21(DE3)plyS containing a specific pTrcHisDUT construct was grown in LB medium containing chloramphenicol (25 μg/ml) and ampicillin (100 μg/ml) at 37°C for 2.5 h. IPTG (1 mM final concentration) was added and the culture was incubated an additional 2 h at 37°C. Bacteria were collected from 1 to 2 liters of medium by low speed centrifugation and the bacterial pellet was resuspended in 50 ml of extraction buffer (50 mM sodium phosphate, 300 mM NaCl and 10 mM imidazole, pH 7.4). Bacteria were lysed by ultrasonication. The resulting homogenate was centrifuged (15,000 × g, 30 min at 4°C), and the supernatant was applied to a HisPur™ spin column, which was equilibrated in extraction buffer. The column was washed three times with two-resin bed volumes of extraction buffer and the dUTPase proteins eluted by washing the column four times with one resin-bed volume of 50 mM sodium phosphate, 300 mM NaCl and 150 mM imidazole, pH 7.4. Fractions were assayed for dUTPase activity as described previously (Glaser et al., 2006) and for protein using the Coomassie Brilliant Blue dye-binding assay (Bio-Rad Laboratories) and bovine serum albumin as the standard. A unit of dUTPase activity was defined as the amount of enzyme required to convert 1 nmole of dUTP to dUMP and pyrophosphate per min at 37°C under the assay conditions. Purity of all recombinant dUTPases (herpesviruses and human encoded) was determined by SDS-PAGE as described previously (Glaser et al., 2006; Ariza and Williams, 2011). Proteins were visualized using EZBlue™ protein gel stain, as described by the manufacturer (Sigma Aldrich, St. Louis, MO). All recombinant herpesviruses and human dUTPase protein preparations were tested for the presence of contaminants as described previously (Glaser et al., 2006; Ariza et al., 2009; Ariza and Williams, 2011; Ariza et al., 2013) and were free of detectable levels of LPS, peptidoglycan (SLP-HS), DNA or RNA. The purified recombinant herpesviruses-encoded and human dUTPase proteins used in these studies were stored at −80°C at stock concentrations of 0.2 and 0.5 mg/ml.

### Cell culture

Human dendritic cells (hDC/LCs; myeloid, plasmacytoid and Langerhan cells) were obtained from MatTek Corporation (Ashland MD). These cells were generated from CD34^+^ progenitor cells derived from human umbilical cord blood (HUCB) cells and cultured using specially formulated medium, DC-100-MM (MatTek), containing a cytokine cocktail designed to induce differentiation of the CD34^+^ into DCs. These DCs express surface markers CD1a, HLA-DR, co-stimulatory molecules, Birbeck granules and surface markers characteristic of both plasmacytoid and myeloid DC (Ayehunie et al., 2003).

Human embryonic kidney 293 (HEK293) cell lines stably expressing either human TLR2 (TLR2-HEK293) or TLR2/TLR6 (TLR2/TLR6-HEK293) as well as control cells (HEK293 WT) were purchased from Invivogen (San Diego, CA). All cell lines were maintained in DMEM supplemented with L-glutamine (2 mM), 4.5 g/l glucose, sodium pyruvate (1%), 10% heat-inactivated FBS, 50 U/ml penicillin, 50 μg/ml streptomycin, 100 μg/ml Normocin™, plus 10 μg/ml blasticidin (TLR2-HEK293; TLR2/TLR6-HEK293).

Human peripheral blood mononuclear cells (PBMCs) from healthy subjects were obtained from Astarte Biologics (Cat# 1001 Lot # 1704OC12).

### Luciferase reporter gene assays

HEK293 cells (TLR2-HEK293, TLR2/TLR6-HEK293 and HEK293 WT) (2.5 × 10^5^) were seeded into 12-well plates and 24 h later transiently transfected using lipofectamine 2000 transfection reagent (Invitrogen; Carlsbad, CA), as we have previously described (Ariza et al., 2009, 2013; Ariza and Williams, 2011). For NF-κB reporter assays, cells were transfected with pNFκ B-Luc (0.5 μ g) and pRL-TK (8 ng) reporter vectors and co-transfected with the expression plasmids pCMV-TLR1, pCMV-MyD88 dominant-negative (MyD88DN), pZero-hTLR2 dominant- negative (TLR2DN) or empty vector (0.3 μ g), as described in the figure legends. All luciferase experiments described in **Figures 2**, **3** were performed simultaneously, repeated three times and empty vector was used as a control/carrier to keep the total amount of transfected-DNA constant. At 24–36 h following transfection, cells were treated with various concentrations (0–10 μg/ml) of purified recombinant dUTPase proteins from HSV-2, HHV-6A, HHV-8 and VZV or left untreated for 8 h. Specific TLR ligands (zymosan: 10 μg/ml; Pam3Csk4: 0.1 μg/ml; and FSL-1: 0.1 μg/ml) were used as positive controls for TLR2, TLR1, and TLR6 (Ariza et al., 2009). Following treatment, cell lysates were prepared and reporter gene activities were measured using the dual-luciferase reporter system (Promega, Madison, WI), as we have described (Ariza et al., 2009; Ariza and Williams, 2011; Ariza et al., 2013). Data were normalized for transfection efficiency by measuring *Renilla* luciferase activity and expressed as the mean relative stimulation ± *SD*.

### Human TLR2 blocking experiments

TLR2-expressing HEK293 cells were transiently transfected with pNFκB-Luc and pRL-TK reporter vectors and co-transfected with the expression plasmid pCMV-TLR1 or empty vector as described above. At 24–36 h after transfection, cells were pretreated with 10 μg/ml of either anti-human TLR2 mAb (anti-TLR2 mAb; clone TL2.1) or IgG2a isotype control Ab for 1 h at 37°C, and subsequently treated with dUTPase proteins (10 μg/ml) encoded by HHV-6A, HHV-8 and VZV or left untreated for 8 h. After treatment, cell lysates were prepared, and neutralization of TLR2-mediated activation of NF-κB reporter gene activity was determined using the dual-luciferase reporter assay as described above (Ariza et al., 2009, 2013; Ariza and Williams, 2011). Data were normalized for transfection efficiency by measuring *Renilla* luciferase activity and expressed as the mean relative stimulation ± *SD*.

### Cytokine profile induced by herpesviruses-encoded dUTPases

hDCs and PBMCs were seeded at a density of 2.5 × 10^5^ in 24-well plates and cultured in AIM-V serum-free medium supplemented with L-glutamine (2 mM), streptomycin (50 μg/ml) and gentamycin (10 μg/ml). The next day, cells were stimulated with the herpesviruses-encoded dUTPases (0.1 or 10 μg/ml), Pam3Csk4 (0.1 μg/ml; as described previously, Ariza et al., 2013), or left untreated for 24 h. Nuclear human dUTPase protein (10 μg/ml) was used as a control. Following treatment, cell culture supernatants were collected and the levels of cytokines in treated and control samples were measured by ELISA (MSD Multi-array and Multi-spot human cytokine kit) as we have described previously (Ariza et al., 2013). Concentrations are expressed as pg/ml and represent the mean ± *SD* of an n of 4.

For blocking experiments, hDCs and PBMCs were seeded at a density of 2.5 × 10^5^ in 24-well plates and cultured in AIM-V serum-free medium supplemented with L-glutamine (2 mM), streptomycin (50 μg/ml) and gentamycin (10 μg/ml). The next day, cells were pretreated with (10 μg/ml) anti-human TLR2 monoclonal antibody (anti-TLR2 MAb; clone TL2.1) or IgG2a MAb isotype control for 1 h at 37°C and subsequently exposed to the herpesviruses-encoded dUTPases (10 μg/ml), Pam3Csk4 (0.1 μg/ml; as described previously, Ariza et al., 2013) or left untreated for 24 h. Following treatment, cell culture supernatants were collected and the levels of pro-inflammatory cytokines in treated and control samples were measured by ELISA as we have described (Ariza et al., 2013). Concentrations are expressed as pg/ml and represent the mean ± *SD* of an n of 4.

### Statistical analysis

Statistical analyses were performed using a paired two-sample *t*-test for the means. For comparison of cytokine production induced by each specific herpesvirus-encoded dUTPase treatment relative to the untreated control, the Student's *t* test was used and *p* values reported when significant (*p* < 0.05). A two-sample *t-test* was also used to compare cytokine levels among groups in the presence or absence of blocking antibody. Values represent the mean ± *SD* of at least three independent experiments.

## Results

### Purification of recombinant his-tagged herpesviruses-encoded dUTPases

**Figure 1:**
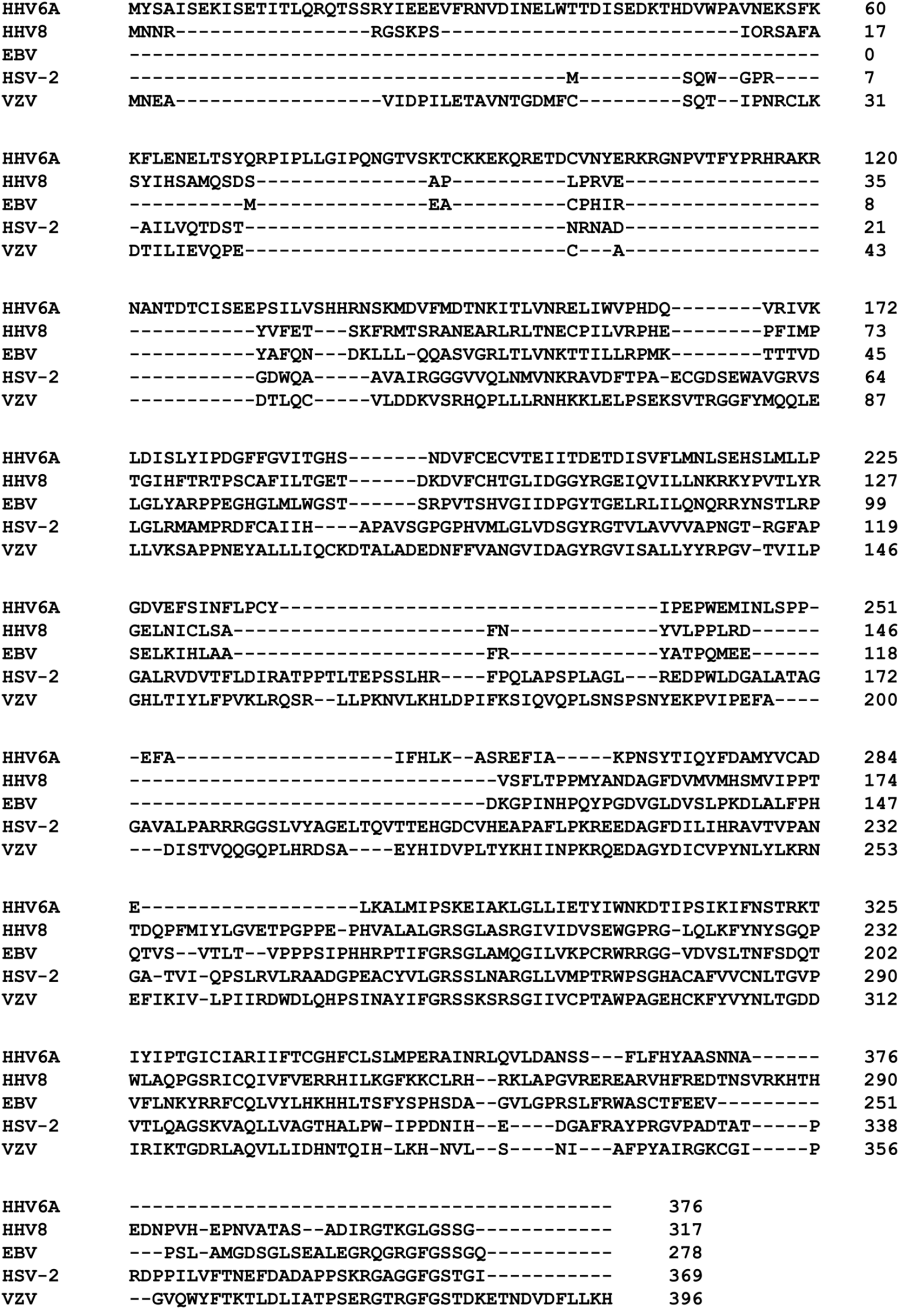
**Herpesviruses-encoded dUTPase proteins**. CLUSTAL Omega (1.2.0) multiple sequence alignment of herpesviruses-encoded dUTPase recombinant proteins used in this study.

The recombinant his-tagged herpesviruses-encoded dUTPase proteins were routinely purified 520–830-fold using HisPur™ affinity chromatography. Molecular weights of the recombinant his-tagged dUTPase proteins based upon SDS-PAGE were 42, 48, 40, and 50 kDa for HSV-2, HHV-6A, HHV-8, and VZV, respectively, which coincide with the molecular weights predicted for these recombinant proteins. Based upon the sensitivity (5 ng) of the EZBlue™ stain, the purified recombinant herpesviruses-encoded dUTPase proteins were estimated to be greater than 99% homogeneous (data not shown). To ensure the high purity of the recombinant dUTPase proteins, all herpesviruses his-tagged recombinant dUTPase preparations were tested as described previously (Glaser et al., 2006; Ariza et al., 2009; Ariza and Williams, 2011) and shown to be peptidoglycan (SLP-HS), DNA/ RNA and endotoxin free (<0.08 IU/ml). With the exception of the HHV-6A recombinant dUTPase protein, which had no detectable enzymatic activity under our assay conditions, all the remaining recombinant his-tagged dUTPase proteins (HSV-2: 10.4 ± 0.53; HHV-8: 5.8 ± 0.23 and VZV: 6.3 ± 0.42 units/mg protein) possessed enzymatic activity specific for dUTP. The protein sequences for the cloned dUTPase genes are shown in Figure 1.

### The herpesviruses-encoded dUTPases induce the transcriptional activation of NFκB via TLR2/TLR1

**Figure 2:**
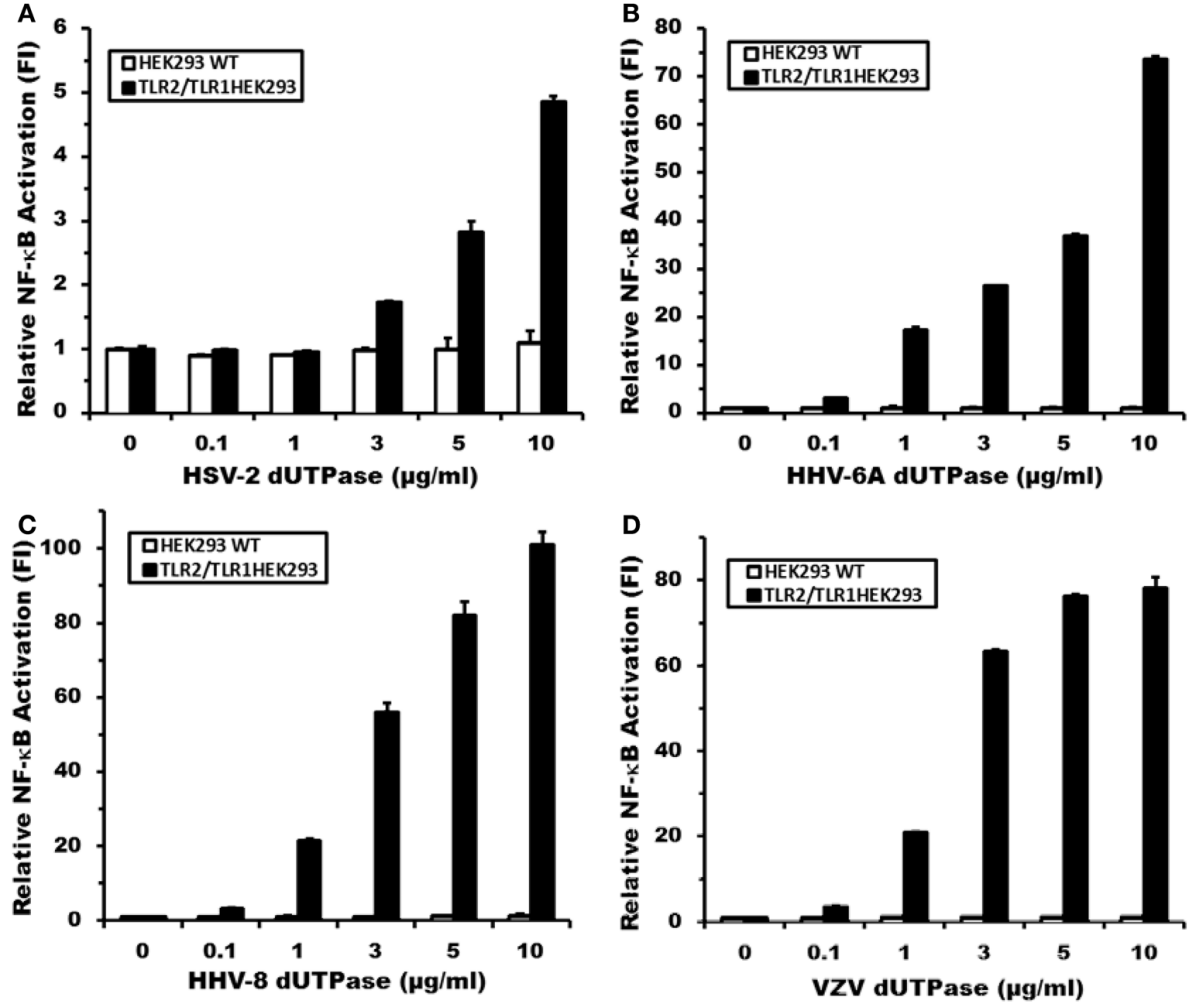
**Herpesviruses-encoded dUTPases induce differential NF-κB activation through TLR2/TLR1 heterodimers**. Dose-response activation of NF-κB by purified recombinant dUTPase proteins from **(A)** herpes simplex virus type 2 (HSV-2), **(B)** human herpesvirus-6A (HHV-6A), **(C)** human herpesvirus-8 (HHV-8), **(D)** varicella-zoster virus (VZV), in TLR2-HEK293 and HEK293 wild-type control cell lines, as determined by luciferase reporter assay. HEK293 cells were transiently transfected with NF-κ B luciferase and pRL-TK reporter plasmids and co-transfected with pCMV-TLR1 (TLR2HEK293 cells), or empty vector (HEK293 WT cells), as described in Materials and Methods and by our group (Ariza et al., 2009, 2013; Ariza and Williams, 2011). Empty vector was used as a carrier to keep the total amount of transfected-DNA constant. After 24–36 h, cells were treated with various concentrations of the herpesviruses-encoded dUTPase proteins (0–10 μg/ml), or left untreated for 8 h and luciferase reporter gene activity was measured. Data were normalized for transfection efficiency by measuring *Renilla* luciferase activity and expressed as the mean fold induction ± *SD* relative to untreated control levels. Values represent the average of three independent experiments.

To determine whether human herpesviruses-encoded dUTPase proteins could activate NF-κB through TLR2 in a similar manner as the EBV-encoded dUTPase, HEK293 cells were transiently transfected with vectors encoding the NF-κB luciferase reporter gene, transfection control pRL-TK and co-transfected with either the expression plasmid pCMV-TLR1 (TLR2-HEK293 cells), or empty pCMV vector (HEK293 WT cells). Empty pCMV vector was used as a carrier to keep the total amount of transfected-DNA constant. TLR2-HEK293 cells transfected with pCMV-TLR1 are referred to from here on as TLR2/TLR1-HEK293. After 24–36 h, cells were stimulated with the human herpesviruses-encoded dUTPases (0–10 μg/ml), or no stimulation for 8 h. Treatment of TLR2/TLR1-HEK293 cells with various concentrations of the herpesviruses-encoded dUTPases resulted in the activation of NF-κB in a dose-dependent manner, ranging from 2 to 101-fold induction (Figures 2A–D) relative to the untreated control. However, treatment of control HEK293 cells with the herpesviruses-encoded dUTPases did not result in the activation of the NF-κB promoter (Figures 2A–D). Interestingly, the highest level of NF-κB activation was induced by HHV-8 followed by HHV-6A and VZV encoded dUTPases with HSV-2-encoded dUTPase (Figure 2A) inducing the lowest level of NF-κB activation.

**Figure 3:**
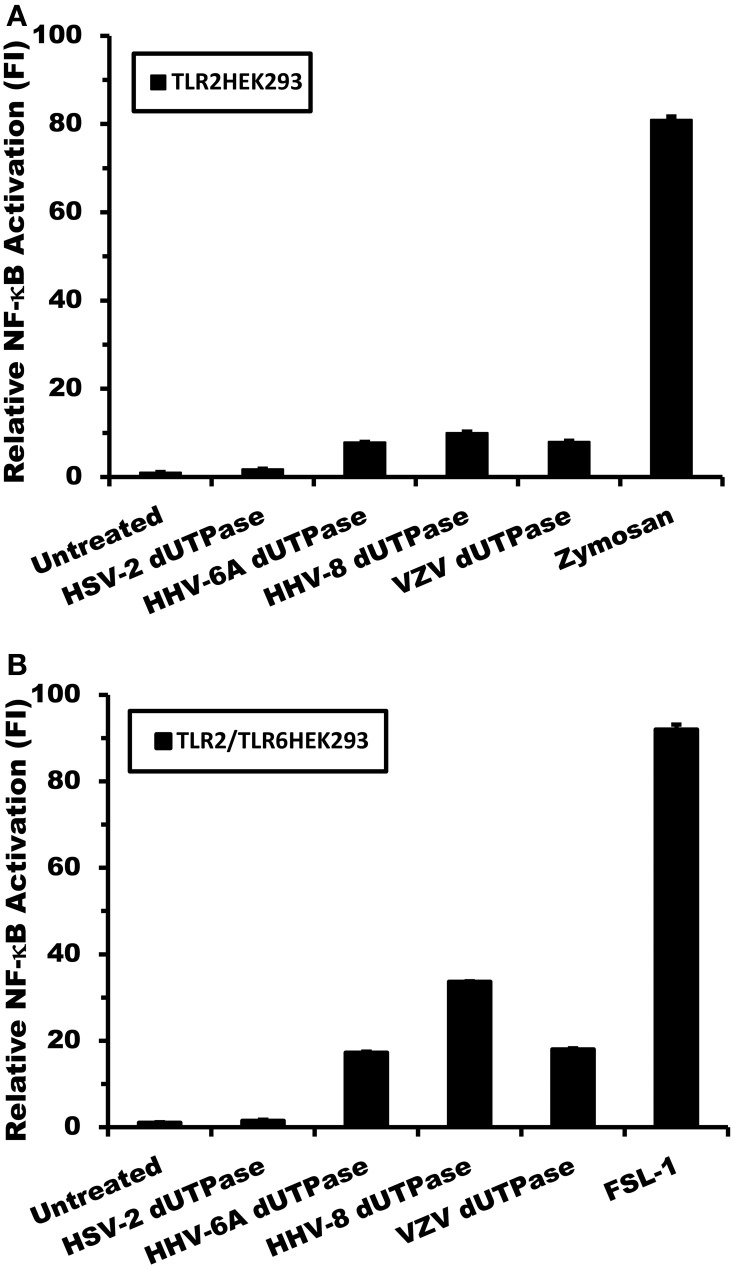
**Activation of NF-κB by herpesviruses-encoded dUTPases is weaker in TLR2/TLR6-HEK293 than in TLR2/TLR1-HEK293 expressing cells**. Herpesviruses-encoded dUTPases mediated activation of NF-κB in **(A)** TLR2-HEK293 and **(B)** TLR2/TLR6-HEK293 cell lines. Cells were transiently transfected with NF-κB luciferase and pRL-TK reporter plasmids and co-transfected with empty vector, as described in Materials and Methods. Empty vector was used as a carrier to keep the total amount of transfected-DNA constant. After 24–36 h, cells were treated with various herpesviruses-encoded dUTPases (10 μg/ml) or left untreated for 8 h, and NF-κB luciferase levels were measured. Zymosan (10 μg/ml) and FSL-1 (0.1 μg/ml) were used as positive controls for TLR2 and TLR6 activation, respectively. Data were normalized for transfection efficiency by measuring *Renilla* luciferase activity and expressed as the mean fold induction ± *SD* relative to control levels. Values represent the average of three independent experiments.

Simultaneously, the TLR2-HEK293 and TLR2/TLR6-HEK293 cell lines were employed to determine whether the activation of NF-κB induced by the herpesviruses-encoded dUTPases involved the formation of TLR2 homodimers or TLR2/TLR6 heterodimers. Cells were transiently transfected with vectors encoding the NF-κB luciferase reporter gene, transfection control pRL-TK and co-transfected with empty vector control, followed by treatment with the herpesviruses-encoded dUTPases (10 μg/ml), zymosan (a TLR2 ligand), FSL-1 (a TLR6 ligand) or no treatment for 8 h, as described in Materials and Methods. As shown in Figure 3A, treatment of TLR2-HEK293 cells with the herpesviruses-encoded dUTPases from HSV-2, HHV-6A, HHV-8, and VZV resulted in either no activation of the NF-κB promoter (HSV-2: 1.1) or a much weaker activation (HHV-6A: 7.8; HHV-8: 10; VZV: 8-fold induction) than that observed in TLR2/TLR1-HEK293 expressing cells treated with the same concentration (HSV-2: 4.5; HHV-6A: 74; HHV-8: 101; VZV: 78-fold induction). Similarly, treatment of TLR2/TLR6-HEK293 cells (Figure 3B) with these herpesviruses-encoded dUTPases (10 μg/ml) induced a higher activation (HHV-6A: 17; HHV-8: 34; VZV: 18-fold induction) of NF-κB than in HEK293 cells just expressing TLR2, but weaker than what was observed in TLR2/TLR1-HEK293 expressing cells (Figures 2B–D). All together, these data demonstrate that the herpesviruses-encoded dUTPases serve as pathogen-associated molecular pattern (PAMP) proteins and are most efficiently recognized by TLR2/TLR1 heterodimers.

**Figure 4:**
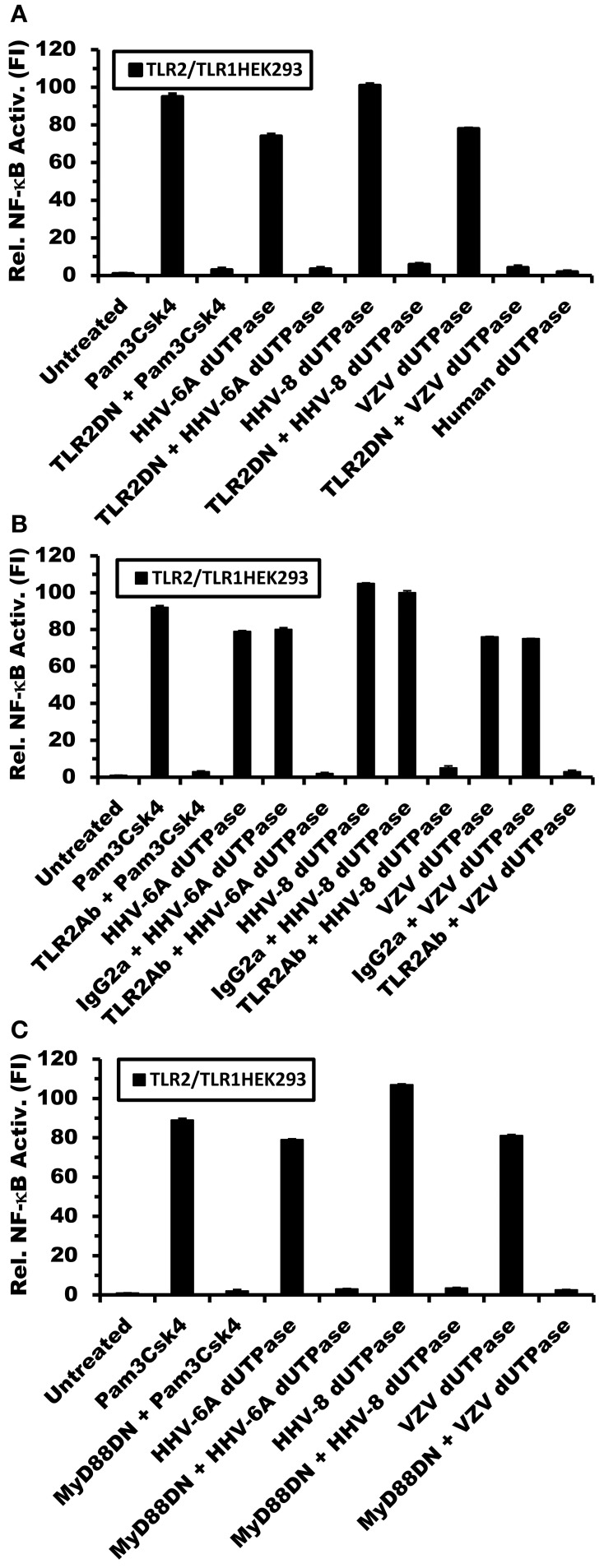
**Herpesviruses-encoded dUTPases signal through TLR2/TLR1 and MyD88**. TLR2- HEK293 cells were transiently transfected with NF-κB luciferase and pRL-TK reporter plasmids and co-transfected with pCMV-TLR1 and either TLR2DN, a vector that expresses a dominant-negative form of TLR2, or empty vector (0.3 μg) **(A)**. After 24–36 h, cells were treated with the dUTPases (10 μg/ml) encoded by HHV-6A, HHV-8 and VZV or left untreated for 8 h, and luciferase reporter gene activity was measured. Pam3Csk4 (0.1 μg/ml) was used as a positive control for TLR2/TLR1 activation and nuclear human dUTPase was used as a control protein. **(B)** Anti-TLR2 blocking Ab inhibits NF-κB activation by the herpesviruses-encoded dUTPases. TLR2-HEK293 cells were transiently transfected with NF-κB luciferase and pRL-TK reporter plasmids and co-transfected with pCMV-TLR1. After 24–36 h, cells were pre-incubated with isotype control (IgG2a) or anti-TLR2 mAbs (10 μg/ml) for 1 h followed by treatment with either herpesviruses-encoded dUTPases (10 μg/ml), Pam3Csk4 for 8 h or left untreated and luciferase reporter gene activity was measured. **(C)** TLR2-HEK293 cells were transiently transfected with NF-κB luciferase and pRL-TK reporter plasmids and co-transfected with pCMV-TLR1 and either MyD88DN, a vector that expresses a dominant-negative form of MyD88, or empty vector. After 24–36 h, cells were treated with either herpesviruses-encoded dUTPases (10 μg/ml), Pam3Csk4 or left untreated for 8 h and luciferase reporter gene activity was measured. Data are expressed as the mean fold induction ± *SD* relative to control levels. Values represent the average of three independent experiments.

To further confirm that the herspesviruses-encoded dUTPases serve as PAMP proteins recognized by TLR2/TLR1, TLR2-HEK293 cells were transfected with pCMV-TLR1 and co-transfected with either a dominant-negative expression plasmid of TLR2 (pZero-hTLR2; [TLR2DN] lacks the TIR domain) or empty vector, followed by treatments with the dUTPases (10 μg/ml) encoded by HHV-6A, HHV-8 and VZV, Pam3Csk4 (0.1 μg/ml) or left untreated for 8 h. Human dUTPase protein (10 μg/ml), was used as a control. As demonstrated in Figure 4A, all herspesviruses-encoded dUTPase recombinant proteins, except the human dUTPase, induced NF-κB activity. However, overexpression of TLR2DN blocked the activation of NF-κB induced by all herpesviruses-encoded dUTPases (HHV-6A, HHV-8, VZV) and Pam3Csk4.

In order to demonstrate conclusively that the herpesviruses-encoded dUTPases are recognized by TLR2/TLR1, we next performed blocking experiments using anti-TLR2 or isotype control Abs. TLR2-expressing HEK293 cells were transiently transfected with the pNF-κB luciferase reporter and pRL-TK plasmids and co-transfected with pCMV-TLR1 expression vector or empty plasmid. After 24–36 h, cells were incubated with anti-TLR2 or IgG2a isotype control Abs (10 μg/ml) for 1 h prior to treatment with the dUTPases encoded by HHV-6A, HHV-8 and VZV or Pam3Csk4 for an additional 8 h. As shown in Figure 4B, while pre-treatment with isotype control Ab had no effect on the ability of the herpesviruses-encoded dUTPases to stimulate NF-κB reporter activity, the anti-TLR2 Ab effectively blocked/inhibited NF-κB activation by these viral dUTPases. These data demonstrate that the activation of NF-κB induced by the herpesviruses-encoded dUTPases is dependent on TLR2/TLR1 heterodimers.

To determine whether the adapter molecule MyD88 was involved in herpesviruses-encoded dUTPases signaling through TLR2/TLR1 heterodimers, TLR2/TLR1-HEK293 expressing cells were co-transfected with either MyD88DN, a plasmid expressing a dominant-negative form of MyD88, or empty vector, followed by treatment with the viral (HHV-6A, HHV-8, VZV) dUTPases (10 μg/ml), Pam3Csk4 (0.1 μg/ml), or left untreated for 8 h. As expected, overexpression of MyD88DN prevented the activation of NF-κB by the herpesviruses-encoded dUTPases and Pam3Csk4 treatments (Figure 4C).

### The herpesviruses-encoded dUTPases induce the secretion of cytokines in human DCs (hDCs) and PBMCs

We have previously demonstrated that the EBV-encoded dUTPase induced the production of pro-inflammatory cytokines in PBMCs, human monocyte derived macrophages and hDCs (Glaser et al., 2006; Waldman et al., 2008; Ariza et al., 2009, 2013). To determine whether the dUTPases encoded by other human herpesviruses induced the production of pro-inflammatory cytokines, ELISA studies were performed in hDCs and PBMCs treated with herpesviruses-encoded dUTPases, control human recombinant dUTPase or left untreated for 24 h, as described in Materials and Methods. Pam3Csk4 (0.1 μg/ml) was used as a control, as we have previously described (Ariza et al., 2013). As shown in Tables 1, 3, 5, treatment of primary hDCs with dUTPase recombinant proteins from HHV-6A, HHV-8 and VZV resulted in a statistically significant (*p* < 0.05) increase in the production of cytokines IL-10, IL12p70, IL-1β, IL-6, IL-8, and TNF-α, even at dUTPase concentrations as low as 0.1 μg/ml. Conversely, only cytokines IL-6 and IL-8 (Table 7) were significantly induced by the HSV-2-encoded dUTPase at the lowest concentration (0.1 μg/ml) tested (*p* < 0.05). Similarly, while all the herpesviruses-encoded dUTPases induced a statistically significant (*p* < 0.05) increase in the production of cytokines in PBMCs at the highest concentration (10 μg/ml) (Tables 2, 4, 6, 8), only the dUTPases encoded by HHV-8 and VZV were capable of inducing a statistically significant (*p* < 0.05) increase in the production of cytokines (Table 4: IFN-γ, IL-1β and TNF-α; and Table 6: IL-10, IL-1β, and IL-8), at the lowest concentration (0.1 μg/ml). Interestingly, the nuclear human dUTPase, which was used as a control protein, did not induce a statistically significant secretion of cytokines even at the highest concentration (10 μg/ml) tested following stimulation of primary DCs (Table 9) or PBMCs (data not shown), consistent with our previously published work (Ariza et al., 2013). These data demonstrate that there are differences in the levels of cytokines induced in response to the various herpesviruses-encoded dUTPases used in this study with HSV-2 exhibiting the lowest cytokine response.

**Table 1:** **Cytokine profile induced by HHV6A-encoded dUTPase in hDCs**.

**Treatments^a^**	**IL-10**	**IL12p70**	**IL-1β**	**IL-6**	**IL-8**	**TNFα**
Untreated	0.64 ± 0.61	1.83 ± 0.84	1.75 ± 0.21	1.79 ± 1.05	304.22 ± 1.31	13.58 ± 1
HHV6AdUTPase (0.1 μg/ml)	10.52 ± 0.8^**^	6.26 ± 0.92^*^	3.91 ± 0.37^**^	44.08 ± 2.96^**^	1523.02 ± 38.47^**^	36.61 ± 5.51^**^
HHV6A-dUTPase (10 μg/ml)	61.63 ± 5.02^**^	32.40 ± 3.46^*^	6.33 ± 0.75^**^	217.48 ± 27.35^**^	9130.50 ± 934.73^**^	177.89 ± 26.66^**^
HHV6A-dUTPase + TLR2 Ab	15.01 ± 0.61^**^	20.55 ± 6.91	2.44 ± 0.12^**^	96.13 ± 7.19^**^	4370.80 ± 43.96^**^	70.14 ± 8.32^*^
HHV6A-dUTPase + Isotype Ctl Ab	58.03 ± 3.45	27.33 ± 3.15	6.09 ± 0.99	207.15 ± 9.12	8130.30 ± 855.04	156.68 ± 21.19

a hDCs were treated with HHV6A-encoded dUTPase (0.1 and 10 μg/ml) or left untreated, as described in Materials and Methods. For blocking experiments, hDCs were pre-incubated with anti-TLR2 or isotype control Abs (10 μg/ml) for 1h and subsequently treated with HHV6A-encoded dUTPase (10 μg/ml), as described in Materials and Methods. After 24 h, culture supernatants were collected for cytokine analysis by ELISA as we have described (Ariza et al., 2009, 2013). Cytokine levels are expressed as pg/ml. Data represent the means ± SD of an n of 4.

* *p < 0.05*,

** *p < 0.01 (Groups compared: dUTPase treated vs. untreated or TLR2 Ab treated vs. isotype control Ab treated samples)*.

**Table 2:** **Cytokine profile induced by HHV6A-encoded dUTPase in PBMCs**.

**Treatments^a^**	**IFN-γ**	**IL-10**	**IL12p70**	**IL-1β**	**IL-6**	**IL-8**	**TNFα**
Untreated	3.75 ± 2.84	0.82 ± 0.67	26.46 ± 5.84	0.56 ± 0.30	29.95 ± 10.93	3910.50 ± 133	58.49 ± 23.45
HHV6A-dUTPase (0.1 μg/ml)	4.01 ± 1.04	0.75 ± 0.19	21.79 ± 1.92	0.50 ± 0.14	30.79 ± 3.47	4021 ± 138	61.47 ± 7.51
HHV6A-dUTPase (10 μg/ml)	322.24 ± 14.38^**^	210.31 ± 2.52^**^	292.65 ± 28.49^**^	38.17 ± 3.56^**^	3571.08 ± 102.51^**^	44,297 ± 523^**^	11,822 ± 125^**^
HHV6A-dUTPase + TLR2 Ab	109.73 ± 28.04^**^	71.52 ± 6.29^**^	54.13 ± 3.86^**^	18.20 ± 3.07^**^	1903.74 ± 227.85^**^	4293 ± 47^**^	7051.25 ± 495.74^**^
HHV6A-dUTPase + Isotype Ctl Ab	342.06 ± 39.50	219.78 ± 9.52	334.29 ± 10.11	33.17 ± 0.42	3701.36 ± 20.04	45,154 ± 1.41	12,214.48 ± 83

a PBMCs were treated with HHV6A-encoded dUTPase (0.1 and 10 μg/ml) or left untreated, as described in Materials and Methods. For blocking experiments, PBMCs were pre-incubated with anti-TLR2 or isotype control Abs (10 μg/ml) for 1 h and subsequently treated with HHV6A-encoded dUTPase (10 μg/ml) or left untreated, as described in Materials and Methods. After 24 h, culture supernatants were collected for cytokine analysis by ELISA as we have described (Ariza et al., 2009, 2013). Cytokine levels are expressed as pg/ml. Data represent the means ± SD of an n of 4.

** *p < 0.01 (Groups compared: dUTPase treated vs. untreated or TLR2 Ab treated vs. isotype control Ab treated samples)*.

**Table 3:** **Cytokine profile induced by HHV8-encoded dUTPase in hDCs**.

**Treatments^a^**	**IL-10**	**IL12p70**	**IL-1β**	**IL-6**	**IL-8**	**TNFα**
Untreated	0.64 ± 0.61	1.83 ± 0.84	1.75 ± 0.21	1.79 ± 1.05	304.22 ± 1.31	13.58 ± 1
HHV8-dUTPase (0.1 μg/ml)	4.70 ± 0.74^**^	5.65 ± 2.69^*^	2.65 ± 0.05^**^	21.86 ± 2.47^**^	1091.73 ± 102.81^**^	27.26 ± 4.70^**^
HHV8-dUTPase (10 μg/ml)	47.83 ± 2^**^	33 ± 1.85^**^	5.46 ± 0.48^**^	157.39 ± 11.17^**^	7716.23 ± 144.95^**^	155.31 ± 12.78^**^
HHV8-dUTPase + TLR2 Ab	8.49 ± 0.6^**^	20.57 ± 1.60^**^	1.74 ± 0.10^**^	73.60 ± 6.07^**^	2229.22 ± 112.71^**^	60.81 ± 5.72^**^
HHV8-dUTPase + Isotype Ctl Ab	45.55 ± 2.11	31.99 ± 5.84	4.81 ± 0.70	160.39 ± 15.18	7025.33 ± 76.36	146.30 ± 7.87

a hDCs were treated with HHV8-encoded dUTPase (0.1 and 10 μg/ml) or left untreated, as described in Materials and Methods. For blocking experiments, hDCs were pre-incubated with anti-TLR2 or isotype control Abs (10 μg/ml) for 1 h and subsequently treated with HHV8-encoded dUTPase (10 μg/ml) or left untreated, as described in Materials and Methods. After 24 h, culture supernatants were collected for cytokine analysis by ELISA as we have described (Ariza et al., 2009, 2013). Cytokine levels are expressed as pg/ml. Data represent the means ± SD of an n of 4.

* *p < 0.05*,

** *p < 0.01 (Groups compared: dUTPase treated vs. untreated or TLR2 Ab treated vs. isotype control Ab treated samples)*.

**Table 4:** **Cytokine profile induced by HHV8-encoded dUTPase in PBMCs**.

**Treatments^a^**	**IFN-γ**	**IL-10**	**IL12p70**	**IL-1β**	**IL-6**	**IL-8**	**TNFα**
Untreated	3.75 ± 2.84	0.82 ± 0.67	26.46 ± 5.84	0.56 ± 0.30	29.95 ± 10.93	3910.50 ± 133	58.49 ± 23.45
HHV8-dUTPase (0.1 μg/ml)	7.07 ± 1.55^*^	1.46 ± 0.40	28.72 ± 2.23	1.14 ± 0.45^*^	37.26 ± 10.06	4080 ± 45	82.37 ± 5.20
HHV8-dUTPase (10 μg/ml)	316.49 ± 3.89^**^	205.15 ± 17.35^**^	316.59 ± 10.37^**^	35.35 ± 4.04^**^	3433.72 ± 116.53^**^	44,780 ± 548^**^	11,935 ± 285^**^
HHV8-dUTPase + TLR2 Ab	123.53 ± 24.35^**^	75.58 ± 2.33^**^	51.02 ± 2.09^**^	14.87 ± 2.19^**^	1771.32 ± 115.78^**^	4207 ± 17^**^	6593.07 ± 300.90^**^
HHV8-dUTPase + Isotype Ctl Ab	304.41 ± 18.35	198.23 ± 0.82	314.21 ± 8.52	38.26 ± 5.64	3278.12 ± 120.79	43,738.17 ± 355.27	12,354.62 ± 306.76

a PBMCs were treated with HHV8-encoded dUTPase (0.1 and 10 μg/ml) or left untreated, as described in Materials and Methods. For blocking experiments, PBMCs were pre-incubated with anti-TLR2 or isotype control Abs (10 μg/ml) for 1 h and subsequently treated with HHV8-encoded dUTPase (10 μg/ml) or left untreated, as described in Materials and Methods. After 24 h, culture supernatants were collected for cytokine analysis by ELISA as we have described (Ariza et al., 2009, 2013). Cytokine levels are expressed as pg/ml. Data represent the means ± SD of an n of 4.

* *p < 0.05*,

** *p < 0.01 (Groups compared: dUTPase treated vs. untreated or TLR2 Ab treated vs. isotype control Ab treated samples)*.

**Table 5:** **Cytokine profile induced by VZV-encoded dUTPase in hDCs**.

**Treatments^a^**	**IL-10**	**IL12p70**	**IL-1β**	**IL-6**	**IL-8**	**TNFα**
Untreated	0.64 ± 0.61	1.83 ± 0.84	1.75 ± 0.21	1.79 ± 1.05	304.22 ± 1.31	13.58 ± 1
VZV-dUTPase (0.1 μg/ml)	13.23 ± 0.24^*^	24.31 ± 2.40^**^	4.91 ± 1.59^*^	94.31 ± 45.08^*^	2231.42 ± 45.43^**^	47.69 ± 16^*^
VZV-dUTPase (10 μg/ml)	76.34 ± 16.11^**^	58.68 ± 22.12^**^	8.28 ± 1.73^**^	192.88 ± 47.91^**^	4665.63 ± 231.7^**^	112.06 ± 29.92^**^
VZV-dUTPase + TLR2 Ab	8.84 ± 0.81^**^	23.76 ± 1.70	2.07 ± 0.14^**^	75.34 ± 9.82^**^	4242.27 ± 59.83^**^	68.74 ± 23.13^*^
VZV-dUTPase + Isotype Ctl Ab	78.41 ± 0.89	56.30 ± 7.66	7.21 ± 0.37	172.12 ± 12.73	4461.11 ± 435.95	104.88 ± 16.29

a hDCs were treated with VZV-encoded dUTPase (0.1 and 10 μg/ml) or left untreated, as described in Materials and Methods. For blocking experiments, hDCs were pre-incubated with anti-TLR2 or isotype control Abs (10 μg/ml) for 1 h and subsequently treated with VZV-encoded dUTPase (10 μg/ml) or left untreated, as described in Materials and Methods. After 24 h, culture supernatants were collected for cytokine analysis by ELISA as we have described (Ariza et al., 2009, 2013). Cytokine levels are expressed as pg/ml. Data represent the means ± SD of an n of 4.

* *p < 0.05*,

** *p < 0.01 (Groups compared: dUTPase treated vs. untreated or TLR2 Ab treated vs. isotype control Ab treated samples)*.

**Table 6:** **Cytokine profile induced by VZV-encoded dUTPase in PBMCs**.

**Treatments^a^**	**IFN-γ**	**IL-10**	**IL12p70**	**IL-1β**	**IL-6**	**IL-8**	**TNFα**
Untreated	3.75 ± 2.84	0.82 ± 0.67	26.46 ± 5.84	0.56 ± 0.30	29.95 ± 10.93	3910.50 ± 133	58.49 ± 23.45
VZV-dUTPase (0.1 μg/ml)	8.28 ± 5.14	1.33 ± 0.41^*^	22.11 ± 1.96	0.92 ± 0.17^*^	32.55 ± 2.41	4120 ± 135^*^	62.62 ± 8.04
VZV-dUTPase (10 μg/ml)	265.55 ± 10.27^**^	176.14 ± 5.90^**^	360.62 ± 34.79^**^	31.60 ± 2.21^**^	2621.20 ± 119.19^**^	46,094 ± 407^**^	12,073.55 ± 386.68^**^
VZV-dUTPase + TLR2 Ab	125.87 ± 39.45^**^	68.85 ± 13.23^**^	53.87 ± 2.50^**^	14.51 ± 1.66^**^	1525.65 ± 181.75^**^	4219 ± 55^**^	5835.86 ± 154.34^**^
VZV-dUTPase + Isotype Ctl Ab	205.73 ± 23.70	189.61 ± 3.21	299.48 ± 20.02	33.49 ± 1.76	2851.90 ± 59.57	44,325 ± 503	11,632.16 ± 173.55

a PBMCs were treated with VZV-encoded dUTPase (0.1 and 10 μg/ml) or left untreated, as described in Materials and Methods. For blocking experiments, PBMCs were pre-incubated with anti-TLR2 or isotype control Abs (10 μg/ml) for 1 h and subsequently treated with VZV-encoded dUTPase (10 μg/ml) or left untreated, as described in Materials and Methods. After 24 h, culture supernatants were collected for cytokine analysis by ELISA as we have described (Ariza et al., 2009, 2013). Cytokine levels are expressed as pg/ml. Data represent the means ± SD of an n of 4.

* *p < 0.05*,

** *p < 0.01 (Groups compared: dUTPase treated vs. untreated or TLR2 Ab treated vs. isotype control Ab treated samples)*.

**Table 7:** **Cytokine profile induced by HSV-2-encoded dUTPase in hDCs**.

**Treatments^a^**	**IL-10**	**IL12p70**	**IL-1β**	**IL-6**	**IL-8**	**TNF α**
Untreated	0.64 ± 0.61	1.83 ± 0.84	1.75 ± 0.21	1.79 ± 1.05	304.22 ± 1.31	13.58 ± 1
HSV-2-dUTPase (0.1 μg/ml)	0.57 ± 0.19	2.13 ± 1.14	2.21 ± 0.17	3.39 ± 0.93^*^	429.36 ± 15.94^**^	14.90 ± 2.88
HSV-2-dUTPase (10 μg/ml)	18.52 ± 0.34^**^	14.68 ± 0.87^**^	5.18 ± 0.62^**^	71.42 ± 4.21^**^	3438.26 ± 155.56^**^	56 ± 3.12^**^

a hDCs were treated with HSV-2-encoded dUTPase (0.1; 10 μg/ml) or left untreated, as described in Materials and Methods. After 24 h, culture supernatants were collected for cytokine analysis by ELISA as we have described (Ariza et al., 2009, 2013). Cytokine levels are expressed as pg/ml. Data represent the means ± SD of an n of 4.

* *p < 0.05*,

** *p < 0.01 (Groups compared: dUTPase treated vs. untreated control)*.

**Table 8:** **Cytokine profile induced by HSV-2-encoded dUTPase in PBMCs**.

**Treatments^a^**	**IFNγ**	**IL-10**	**IL12p70**	**IL-1β**	**IL-6**	**IL-8**	**TNFα**
Untreated	3.75 ± 2.84	0.82 ± 0.67	26.46 ± 5.84	0.56 ± 0.30	29.95 ± 10.93	3910.50 ± 133	58.49 ± 23.45
HSV-2-dUTPase (0.1 μg/ml)	3.89 ± 2.24	0.80 ± 0.44	20.29 ± 1.53	0.54 ± 0.22	21.31 ± 1.54	3906 ± 163	47.92 ± 11.63
HSV-2-dUTPase (10 μg/ml)	141.64 ± 29.58^**^	81.06 ± 16.33^*^	78.11 ± 40.70	19.52 ± 2.84^**^	1604.69 ± 153^*^	4398 ± 36	4530.05 ± 842^**^

a PBMCs were treated with HSV-2-encoded dUTPase (0.1 and 10 μg/ml) or left untreated, as described in Materials and Methods. After 24 h, culture supernatants were collected for cytokine analysis by ELISA as we have described (Ariza et al., 2009, 2013). Cytokine levels are expressed as pg/ml. Data represent the means ± SD of an n of 4.

* *p < 0.05*,

** *p < 0.01 (Groups compared: dUTPase treated vs. untreated control)*.

**Table 9:** **Cytokine profile induced by nuclear human-encoded dUTPase in hDCs**.

**Treatments^a^**	**IL-10**	**IL12p70**	**IL-1β**	**IL-6**	**IL-8**	**TNF α**
Untreated	0.47 ± 0.35	1.5 ± 0.47	1 ± 0.15	9.4 ± 1.4	482.7 ± 22.50	7 ± 0.65
Human-dUTPase (10 μg/ml)	2.6 ± 0.2	5.1 ± 2.6	3.6 ± 0.56	96.43 ± 53	760.02 ± 38.47	18.2 ± 2.5

a *hDCs were treated with nuclear human-encoded dUTPase (10 μg/ml) or left untreated, as described in Materials and Methods. After 24 h, culture supernatants were collected for cytokine analysis by ELISA as we have described (Ariza et al., 2009, 2013). Cytokine levels are expressed as pg/ml. Data represent the means ± SD of an n of 4*.

### Human herpesviruses-encoded dUTPases induce cytokines in human primary DCs and PBMCs in a TLR2-dependent manner

To determine whether herpesviruses-encoded dUTPases mediated induction of cytokines was TLR2 dependent, blocking experiments were performed. hDCs and PBMCs were incubated with anti-TLR2 or isotype control (IgG2a) antibodies (10 μg/ml) for 1 h, followed by treatment with specific viral dUTPases encoded by HHV-6A, HHV-8 and VZV or left untreated for 24 h, as we have described (Ariza et al., 2009, 2013). Pam3Csk4 (0.1 μg/ml) was used as a control, as we have previously described (Ariza et al., 2013). After 24 h, culture supernatants from control and treated samples were collected and analyzed for cytokine levels by ELISA. Treatment of hDCs and PBMCS with anti-TLR2 antibody resulted in a statistically significant (*p* < 0.05) decrease in the production of cytokines from hDCs and PBMCs treated with dUTPases encoded by HHV-6A (Tables 1, 2), HHV-8 (Tables 3, 4), and VZV (Tables 5, 6), respectively. However, pre-incubation of cells with the isotype control antibody did not inhibit the secretion of cytokines induced by herpesviruses-encoded dUTPases in PBMCs or hDCs.

## Discussion

The human herpesviruses are a diverse group of organisms exhibiting different cellular tropisms and they are associated with several diseases that have significantly different clinical presentations and outcomes. However, these viruses share several common features such as transmission, which involves contact with host mucosa where they interact with resident cells, including macrophages and DCs, and establish persistent infections. DCs and macrophages are professional antigen presenting cells (APC) that have key roles in regulating immune responses to host pathogens. Considering the roles that these cells have in initiating an immune response against pathogens and the ability of the human herpesviruses to establish persistent infections, it is likely that these viruses have developed mechanism(s) to modulate the functions of DCs and macrophages. While these cell types are not considered to be the primary targets for infection by human herpesviruses, there is accumulating evidence demonstrating that these viruses infect monocytes, macrophages and DCs resulting in non-productive, productive or latent infections (Kondo et al., 1991; Blasig et al., 1997; Hahn et al., 1998; Savard et al., 2000; Abendroth et al., 2001; Mikloska et al., 2001; Zhang et al., 2001; Kakimoto et al., 2002; Li et al., 2002; Morrow et al., 2003; Senechal et al., 2004; Smith et al., 2005; Rappocciolo et al., 2006; Walling et al., 2007; Huch et al., 2010; Goldwich et al., 2011; Wang et al., 2012), in addition to altering their functions (Kruse et al., 2000; Niiya et al., 2004; West et al., 2011; Gregory et al., 2012; Gustafsson et al., 2013; Stefanidou et al., 2013). With the exception of the γ34.5 protein, which has been reported to interfere with DC maturation during HSV-1 infections (Jin et al., 2009; West et al., 2011; Gobeil and Leib, 2012), virus-encoded macromolecules that modulate DC/macrophage maturation and function remain unknown. Another common characteristic of these viruses is their ability to induce the increased secretion of various pro-inflammatory cytokines/chemokines that are either host-derived or virus-encoded, which contribute to the pathophysiology of the virus-associated disease (Compton et al., 2003; Kurt-Jones et al., 2004; Aravalli et al., 2005; Wang et al., 2005; Glaser et al., 2006; Waldman et al., 2008; Ariza et al., 2009, 2013; Gregory et al., 2012). Several studies have demonstrated that the stimulation of TLR2 on monocytes, macrophages or DCs by intact virions or in some cases by herpesvirus-encoded glycoproteins results in the increased secretion of pro-inflammatory cytokines (Compton et al., 2003; Kurt-Jones et al., 2004; Aravalli et al., 2005; Wang et al., 2005; Boehme et al., 2006; Sato et al., 2006; Gaudreault et al., 2007; Paludan et al., 2011; Cal et al., 2012; Leoni et al., 2012).

Recent studies by our group as well as others have shown that the dUTPases encoded by the gamma herpesviruses possess novel functions independent of their enzymatic activity (Glaser et al., 2006; Waldman et al., 2008; Ariza et al., 2009, 2013; Leang et al., 2011; Madrid and Ganem, 2012). Our studies with EBV were the first to demonstrate that the EBV-encoded dUTPase possesses novel functions in innate and adaptive immunity due in part to the activation of TLR2, NF-κB and the subsequent modulation of downstream genes involved in cytokine-receptor signaling pathways, type I/II interferons (IFN) production and effector T-cell function (Glaser et al., 2006; Waldman et al., 2008; Ariza et al., 2009, 2013). The primary cellular targets of the EBV-encoded dUTPase appear to be monocytes and dendritic cells (Glaser et al., 2006; Waldman et al., 2008; Ariza et al., 2009, 2013). Studies with murine γ-herpesvirus-68 (γ-68) have shown that the γ-68-encoded dUTPase (ORF54) possesses anti-interferon properties and is necessary for efficient replication of the virus in the lungs of infected mice (Leang et al., 2011). Madrid and Ganem (2012) reported that the HHV-8- encoded dUTPase, but not the EBV-encoded dUTPase, down-regulated the expression of NKp44L, a “cytotoxicity” receptor on natural killer (NK) cells, suggesting that the HHV-8-encoded dUTPase was involved in immune evasion. However, studies on the potential role of the herpesviruses-encoded dUTPases in pathogenesis have been limited. Furthermore, there have not been any studies to address the role of dUTPases-encoded by the alpha or beta herpesviruses as possible immune modulators. Several studies have demonstrated the expression of the EBV- and HHV-8-encoded dUTPases in human malignancies associated with these viruses (Kremmer et al., 1999; Fleischmann et al., 2002), and anti-dUTPase antibodies have been detected in the serum of patients with several EBV-associated diseases (Fleischmann et al., 2002). Most importantly, we have demonstrated that the presence of antibodies to the EBV-encoded dUTPase may be useful in diagnosing a subset of patients with chronic fatigue syndrome (Lerner et al., 2012) as well as some patients with acute myocardial infarction (Binkley et al., 2013), which further support a role for the EBV-encoded dUTPase in these processes.

Since herpesviruses-encoded dUTPases lack consensus secretory sequences, it has been suggested that the release of these proteins could only occur as a result of cell lysis following lytic replication of the virus. However, there is accumulating evidence, especially in the case of the gamma herpesviruses (Laichalk and Thorley-Lawson, 2005; Al Tabaa et al., 2009, 2011; Myoung and Ganem, 2011; Scholz et al., 2013; Strong et al., 2013), supporting the premise that “*in vivo* abortive-lytic replication” is the predominant type of replication occurring in infected cells, which results in the production of immediate early and early proteins, but limited production of new virions. In addition, it has been demonstrated that abortive replication of human immunodeficiency virus (HIV-1) induces pyroptosis (Doitsh et al., 2014; Monroe et al., 2014). Unlike apoptosis, cell death by pyroptosis results in membrane rupture and release of cytosolic components and macromolecules, which may activate innate immune signaling pathways leading to the secretion of inflammatory cytokines (Sangiuliano et al., 2014; Upton and Chan, 2014). Since IL-1β is a key driver of pyroptosis and this cytokine is induced by the herpesviruses-encoded dUTPases studied, it is possible that pyroptosis may represent a potential mechanism by which these dUTPases can be released from infected cells.

An alternative mechanism to explain the release of herpesviruses-encoded dUTPases, which does not require cell lysis, is through microvesicles/exosomes. There is considerable evidence demonstrating that several herpesviruses, the gamma herpesviruses in particular, modify cellular exosomal proteins (Meckes et al., 2013). Furthermore, viral-encoded macromolecules are also secreted using this pathway (Flanagan et al., 2003; Keryer-Bibens et al., 2006; Meckes et al., 2010; Pegtel et al., 2010). In line with this premise, the human nuclear isoform of the dUTPase has been shown to be secreted in B-cell derived exosomes (Buschow et al., 2010). Furthermore, we recently demonstrated that the EBV-encoded dUTPase is secreted in exosomes during abortive-lytic replication and that these dUTPase-containing exosomes induced a T_H_1/T_H_17 cytokine response that was TLR2-dependent (Ariza et al., 2013). Interestingly, the human adenovirus type 9 E4-ORF1, which encodes for an ancestral dUTPase (Weiss et al., 1997), is also targeted to membrane vesicles (Chung et al., 2007). While there is exciting accumulating literature demonstrating exosomes as a mechanism for the release of several dUTPases of viral and human origin, it is not known whether the dUTPases encoded by other human herpesviruses are released in exosomes during lytic/abortive-lytic replication of these viruses.

The data presented in this study demonstrate that the dUTPases encoded by various human herpesviruses differentially activate NF-κB and induce the secretion of pro-inflammatory cytokines in a TLR2-dependent mechanism. Interestingly, the data also demonstrate that the herpesviruses-encoded dUTPases exhibit different TLR2 partners/complex formation preferences, which influence the levels of NF-κB activation induced by these viral dUTPases, as shown in Figures 2, 3. We have previously reported that the maximal activation of NF-κB by the EBV-encoded dUTPase is independent of TLR1 and TLR6 and requires TLR2 homodimerization (Ariza et al., 2009). Conversely, this study demonstrates rather conclusively that activation of NF-κB by the HHV-6A, HHV-8, and VZV-encoded dUTPases occurs by engaging TLR2/TLR1 heterodimers. This finding was further confirmed by data demonstrating that activation of NF-κB by the herpesviruses-encoded dUTPases is inhibited by TLR2 or MyD88 dominant-negative mutants and anti-hTLR2 but not isotype control Abs (Figure 4). Finally, while the dUTPases encoded by HHV-8, HHV-6A, and VZV were very effective at stimulating NF-κB activity in TLR2 expressing HEK293 cells co-transfected with pCMV-TLR1, the HSV-2-encoded dUTPase was the least effective at all the concentrations tested.

These differences most likely reflect differences in intrinsic properties of each viral protein including binding affinities of the dUTPases to TLR2 and the overall structure of the dUTPases. Proteins are classified as dUTPases based primarily on their enzymatic activity and, in the case of homotrimeric and monomeric dUTPases, the presence of five conserved domains, which are essential for the catalytic site of the enzyme (Baldo and McClure, 1999; McGeehan et al., 2001; Davidson and Stow, 2005). There is little homology between these viral dUTPases except in the five conserved domains involved with enzymatic activity, which are lacking in all members of the β-herpesvirus family (Baldo and McClure, 1999; McGeehan et al., 2001; Davidson and Stow, 2005). We have previously demonstrated, in the case of EBV- and human endogenous retrovirus K (HERV-K-encoded dUTPases, that enzymatic activity is not required for modulating the immune response (Glaser et al., 2006; Waldman et al., 2008; Ariza et al., 2009, 2013; Ariza and Williams, 2011). This finding has now been further confirmed by the data presented in this study demonstrating that like the human cytomegalovirus (HCMV)-encoded dUTPase (Caposio et al., 2004), the HHV-6A-encoded dUTPase lacks functional dUTPase hydrolytic activity. However, the HHV-6-encoded dUTPase is capable of stimulating DCs and PBMCs through TLR2/TLR1 and induce the secretion of cytokines. The size and sequence homologies of the herpesviruses-encoded dUTPase proteins vary greatly; the EBV-encoded dUTPase is the smallest (278 amino acids) while the VZV-encoded dUTPase is the largest (396 amino acids). The greatest identity (26.8%) occurs between the EBV- and HHV-8-encoded dUTPases, and the least identity (10.9%) occurs between the EBV- and HHV-6A-encoded dUTPases. While the crystal structure of several homotrimeric dUTPases, including *Escherichia coli*, human, HERV-K, feline immunodeficiency virus and equine infection anemia virus, has been determined (Cedergren-Zeppezauer et al., 1992; Mol et al., 1996; Dauter et al., 1999; Harris et al., 1999; Prasad et al., 2000), the only structure of monomeric dUTPases that has been determined is for EBV (Tarbouriech et al., 2005). Preliminary studies using blast alignments and Kyte-Doolittle hydropathy analyses have identified a region within a β-hairpin loop that is conserved among these proteins, which may interact with TLR2 and studies are presently underway to address this possibility.

It has been suggested that the herpesvirus dUTPase gene was captured from a host followed by gene duplication and gene fusion (Baldo and McClure, 1999; McGeehan et al., 2001). Over time, additional residues were lost resulting in the formation of proteins lacking enzymatic activity but that possess other biological functions. Analysis of sequence similarities coupled with structural predictions led Davidson and Stow (2005) to suggest that several herpesvirus genes, including the UL31, UL82, UL83, and UL84 genes of HCMV as well as ORF10 and ORF11 of HHV-8, may have been derived from an ancestral herpesvirus dUTPase gene. Interestingly, the UL83 gene product pp65, a structural protein, has been reported to decrease the interferon response (Browne and Shenk, 2003; Abate et al., 2004) and to down-regulate MHC class II protein expression (Wiertz et al., 2007). This phenomenon of an ancestral dUTPase gene contributing to the formation of a protein with novel functions is not limited to the herpesviruses. The human adenovirus type 9 E4-ORF1 gene, which evolved from an ancestral avian dUTPase (Weiss et al., 1997), encodes for a protein that possesses oncogenic properties (Weiss et al., 1997), induces cellular glucose uptake (Dhurandhar et al., 2011) and adipogenesis (Rogers et al., 2008). Likewise, studies by Abergel et al. (1999) suggest that an ancestral dUTPase gene evolved into the primate CD4 and chemokine receptor interacting region of the human immunodeficiency virus glycoprotein 120 (gp120). Gp120 is shed from the mature virion *in vitro*, has been detected in the plasma of patients early during the infection and correlated with higher levels of TNF-α, IL-6, IL-10, INF-α, and IFN-γ (Rychert et al., 2010). Interestingly, this gp120-mediated process has been suggested to contribute to the immune dysfunction during early HIV infection (Rychert et al., 2010) and has been implicated in HIV-1 associated inflammation (Nazli et al., 2010; Kaushic, 2011; Shah et al., 2011). Furthermore, the gp120 has been reported to activate TLR2, and trigger inflammatory cytokine production through NF-κB, suggesting that this is a mechanism by which gp120 could directly initiate innate immune activation (Nazli et al., 2013). While additional studies are required to demonstrate that the biological properties of these proteins are due to the dUTPase motifs, these results further support the hypothesis that dUTPase proteins, especially viral-encoded dUTPases, may have undiscovered functions that modulate various physiological processes, including those involved in host immune responses.

In summary, our results demonstrate that the dUTPases encoded by several human herpesviruses induced a dose-dependent increase in the activation of NF-κB and the secretion of several pro-inflammatory cytokines, which may contribute to the pathophysiology associated with diseases caused by these viruses. Interestingly, TLR2 blocking studies revealed that the enhanced cytokine secretion is herpesviruses-encoded dUTPase-mediated and TLR2-dependent. More importantly, however, is that the data reported in this study, as well as our previous studies on the EBV- and HERV-K-encoded dUTPases (Glaser et al., 2006; Waldman et al., 2008; Ariza et al., 2009, 2013; Ariza and Williams, 2011), demonstrate that some virus-encoded dUTPases possess novel functions as modulators of innate immune responses through TLR2 leading to the activation of NF-κB and cytokine/chemokine-receptor signaling pathways, which may contribute to the inflammatory microenvironment leading to pathogenesis. Finally, this hypothesis is further supported by the revelation that some genes containing ancestral dUTPase motifs encode for proteins that also have immune modulating activities. These findings suggest that viral-encoded dUTPases may represent a novel class of proteins that could be used as targets for the development of novel therapeutics.

## Author contributions

Conceived and designed the experiments: Maria Eugenia Ariza and Marshall V. Williams; Performed the experiments: Maria Eugenia Ariza and Marshall V. Williams; Analyzed the data: Maria Eugenia Ariza and Marshall V. Williams; Wrote the paper: Maria Eugenia Ariza and Marshall V. Williams; Edited the paper: Maria Eugenia Ariza, Marshall V. Williams and Ronald Glaser.

### Conflict of interest statement

The authors declare that the research was conducted in the absence of any commercial or financial relationships that could be construed as a potential conflict of interest.
